# CD14^bright^CD16+ intermediate monocytes are induced by interleukin-10 and positively correlate with disease activity in rheumatoid arthritis

**DOI:** 10.1186/s13075-016-1216-6

**Published:** 2017-02-10

**Authors:** Masako Tsukamoto, Noriyuki Seta, Keiko Yoshimoto, Katsuya Suzuki, Kunihiro Yamaoka, Tsutomu Takeuchi

**Affiliations:** 1 Division of Rheumatology, Department of Internal Medicine, Keio University School of Medicine, 35 Shinanomachi, Shinjuku-ku, Tokyo, 160-8582 Japan; 2 Department of Internal Medicine, Tokyo Dental College Ichikawa General Hospital, 5-11-13 Sugano, Ichikawa, Chiba 272-8513 Japan; 3 Clinical and Translational Research Center, Keio University Hospital, 35 Shinanomachi, Shinjuku-ku, Tokyo, 160-8582 Japan

**Keywords:** Rheumatoid Arthritis, Monocytes, Interleukin-10, CD16

## Abstract

**Background:**

Three different subsets of circulating human monocytes, CD14^bright^CD16- (classical), CD14^bright^CD16+ (intermediate), and CD14^dim^CD16+ (non-classical) have been recently identified. It has been reported that CD14^bright^CD16+ monocytes are increased in rheumatoid arthritis (RA). However, the role of each monocyte subset in the pathogenesis of RA is still unclear. The purpose of this study was to investigate the association of CD14^bright^CD16+ monocytes with RA.

**Methods:**

The study enrolled 35 patients with RA and 14 healthy volunteers. The three subsets of peripheral blood monocytes were analyzed by flow cytometry. Serum cytokines were measured at baseline in patients with RA and in healthy volunteers. CD14^bright^CD16- monocytes were isolated and cultured in vitro with different cytokines for 14 hours, and CD16 induction was assessed.

**Results:**

The proportion of CD14^bright^CD16+ monocytes, and serum interleukin (IL)-6, IL-8, and IL-10 were increased in patients with RA compared to healthy controls. The proportion of CD14brightCD16+ monocytes correlated with the disease activity of RA positively, whereas the proportion of CD14^bright^CD16- monocytes correlated negatively. When isolated CD14^bright^CD16- monocytes were stimulated with IL-6, IL-8, and IL-10, the only cytokine that significantly induced CD16 expression on the cells was IL-10.

**Conclusions:**

The proportion of CD16^bright^CD14+ monocytes was positively correlated with RA disease activity. The expression of CD16 in monocytes was induced by IL-10 but not IL-6, and IL-8 was enhanced in the sera of patients with RA. Our results suggest that CD16^bright^CD14+ monocytes are involved in the pathogenesis of RA and that IL-10 is a key cytokine that regulates CD16 expression in monocytes.

**Electronic supplementary material:**

The online version of this article (doi:10.1186/s13075-016-1216-6) contains supplementary material, which is available to authorized users.

## Background

Rheumatoid arthritis (RA) is a systemic, autoimmune, and chronic inflammatory disease which causes pain and dysfunction and leads to the destruction of joints [1]. The major inflammatory tissue is the synovium, the thin tissue that lines the joint. Immune cells, such as neutrophils, lymphocytes, and monocytes, produce inflammatory cytokines including interleukin (IL)-1, tumor necrosis factor-α (TNF-α), IL-6, and granulocyte-macrophage colony-stimulating factor (GM-CSF) [2], and are involved in the development of inflammation. It is well-known that peripheral blood monocytes are derived from precursors in the bone marrow, migrate into synovial tissue, and differentiate into macrophages that produce pro-inflammatory cytokines [3]. Macrophages involved in synovial inflammation transform into osteoclasts, which cause joint destruction in RA [2, 4]. Osteoclasts are also derived from CD14+ monocytes under the influence of pro-inflammatory cytokines [5]. Circulating CD14+ monocytes serve as precursors of endothelial cells and contribute to the process of angiogenesis in the tissue [6]. Based on this background, the identification and classification of peripheral monocytes responsible for the disease is indispensable to understanding the pathogenesis of RA.

CD16 is a receptor for immunoglobulin (Ig) gamma Fc region III (FcγRIII). Fcγ receptors (FcγRs) are expressed on most of the cells involved in the immune system, including circulating monocytes, and they regulate immune responses through interaction with antibodies [7]. According to these previous studies, immune complexes formed by anti-cyclic citrullinated peptide antibody (ACPA) and citrullinated peptide seem to bind to FcγRs and stimulate immune cell activation and release of inflammatory cytokines in patients with RA [8]. Therefore, FcγRIII is one of the molecules possibly associated with the pathogenesis of RA. It has been reported that aberrant expression of FcγRIII, or the presence of allelic variants, can contribute to the pathogenesis of RA [9]. However, the regulatory mechanisms of FcγRIII in RA are not fully understood.

Until now, circulating human monocytes have been classified into CD14+ CD16- (classical) and CD14+ CD16+ subsets according to their expression levels of CD14 and CD16 [10, 11]. It had been reported that CD14+ CD16+ monocytes are increased in patients with RA [12]. Recently, a new third monocyte subpopulation, CD14^bright^CD16+ monocytes, was defined. According to the new classification system, the CD14+ CD16+ population is classified into CD14^bright^CD16+ (intermediate) and CD14^dim^CD16+ (non-classical) monocytes, depending on the level of CD14 expression [13]. The CD14^bright^CD16- (classical) monocyte is the major subset, while the CD14^bright^CD16+ and CD14^dim^CD16+ subsets occur in lower numbers than classical monocytes [11].

It has been shown that the CD14^bright^CD16+ monocyte population increases in inflammatory or infectious conditions and, upon lipopolysaccharide stimulation [14, 15], produces TNF-α, IL-1β, IL-6, and IL-10. This newly classified CD14^bright^CD16+ monocyte population has been reported to be increased in patients with RA, whereas the CD14^dim^CD16+ monocyte population is not increased [14]. It has been suggested that CD14^bright^CD16+ monocytes may migrate into the synovium from peripheral blood and differentiate into M1 or M2 macrophages in the tissue [16]. However, the role of each subset in RA has not been fully clarified.

In this study, we sought to investigate the involvement of CD14^bright^CD16+ monocytes in the pathogenesis of RA, and possible mechanisms of the enhanced expression of CD16 on monocytes in patients with RA.

## Methods

### Subjects and study design

Patients with RA (*n* = 35) (mean age ± SD 59.8 ± 12.6 years, 82.9% female) who met the 2010 American College of Rheumatology/European League Against Rheumatism Classification criteria, and 14 healthy volunteers (mean age 49.2 ± 10.8 (range 30–72), 12 female) were enrolled into the study. All patients visited Keio University Hospital between January 2013 and May 2014 and had never been treated with methotrexate (MTX) or biological agents. They were considered to have moderate or high disease activity (scoring ≥3.2 on the 28-joint disease activity score based on erythrocyte sedimentation rate (DAS28-ESR). All participants gave written informed consent in accordance with the Declaration of Helsinki. MTX was initiated at an oral dose of 4–16 mg weekly. Monocyte subsets from peripheral blood samples were taken at baseline and after 12 weeks of MTX treatment in the patients. Clinical parameters including C-reactive protein (CRP), ESR, matrix metalloproteinase-3 (MMP-3), ACPA, and rheumatoid factor (RF) titers were obtained by routine clinical laboratory methods. DAS28-ESR scores, DAS28-CRP score, clinical disease activity index (CDAI), and simplified disease activity index (SDAI) were also determined at baseline and after 12 weeks of MTX treatment. Clinical characteristics of the patients were retrospectively collected from their medical records.

### Monocyte subset determination

Heparinized whole blood was stained with phycoerythrin-Cy7 (PE-Cy7)-conjugated anti-CD14 (clone M5E2, BD Pharmingen, San Diego, CA, USA) and V450-conjugated anti-CD16 antibodies (clone 3G8, BD Horizon, San Jose, CA, USA), and analyzed using a flow cytometer with built-in software (MACSQuant Analyzer® and MACSQuantify® software, Miltenyi Biotec, Bergisch Gladbach, Germany). Monocyte subsets were identified on the basis of forward scatter/side scatter characteristics and CD14-positive gating. Subpopulations of CD14^bright^CD16-, CD14^bright^CD16+, and CD14^dim^CD16+ monocytes were distinguished by their surface expression pattern of CD14 and CD16 according to a previous report [11] and the proportion of each monocyte subset was determined.

### Serum immunoassays

Serum samples were collected at baseline and stored at -80 °C. Serum levels of GM-CSF, interferon-γ (IFN-γ), IL-1β, IL-10, IL-12p70, IL-2, IL-6, IL-8, and TNF-α were measured by multiplex electrochemiluminescence assay (Meso Scale Discovery SECTOR Imager 2400 platform®, Meso Scale Discovery, Rockville MD, USA). Serum macrophage colony-stimulating factor (M-CSF) levels were assessed by enzyme-linked immunosorbent assay (ELISA) (Quantikine® ELISA, Human M-CSF Immunoassay, R&D Systems Inc., Minneapolis MN, USA) and calculated using the manufacturer’s software. Values are expressed in pg/mL and presented as median with interquartile range (IQR).

### Stimulation of peripheral monocytes in vitro

Peripheral blood mononuclear cells (PBMCs) were isolated from five healthy volunteers by density gradient centrifugation (Ficoll-Paque®, GE Healthcare, Uppsala, Sweden). To isolate monocyte subsets, cells were stained with phycoerythrin (PE)-conjugated anti-CD14 (clone MφP9, BD Pharmingen) and BV421-conjugated anti-CD16 antibodies (clone 3G8, BD Horizon) and sorted according to their CD14/CD16 expression using a cell sorter (BD Aria III®, BD Biosciences, San Jose CA, USA). CD14^bright^CD16- monocytes were cultured at 2.5 × 10^5^/500 μL in Roswell Park Memorial Institute medium (RPMI-1640®, ATCC, Manassas, VA, USA) with 10% heat-inactivated fetal bovine serum (MP Biomedicals, Santa Ana CA, USA). They were then stimulated for 14 hours with either 100 ng/mL M-CSF, 1-100 (1, 10, 25, 50, or 100) ng/mL IL-10, 100 ng/mL IL-6, or 20 ng/mL IL-8 at 37 °C in a humidified atmosphere containing 5% CO_2_. The stimulated monocytes were stained with PE-conjugated anti-CD14, BV421-conjugated anti-CD16, and allophycocyanin (APC)-conjugated anti-HLA (human leukocyte antigen)-DR (clone LN3, eBioscience, San Diego CA, USA) antibodies and we analyzed the proportion of CD16+ monocytes using a flow cytometer.

### Detection of IL-10 receptor expression

Heparinized whole blood cells from four healthy volunteers were stained with PE-Cy7-conjugated anti-CD14 (clone M5E2, BD Pharmingen), V450-conjugated anti-CD16 (clone 3G8, BD Horizon), and APC-conjugated anti-IL-10 receptor antibodies (clone 3F9, Biolegend, San Diego CA, USA), and the expression levels of IL-10 receptor on the CD14^bright^CD16- monocyte subset was evaluated.

### IL-10 neutralization assay

CD14^bright^CD16- monocytes (2.5 × 10^5^/500 μL) from the peripheral blood of four healthy volunteers were incubated with 25 ng/mL IL-10. In some experiments, anti-IL-10 receptor antibody (5 μg/mL) (clone 3F9, Biolegend) or rat IgG2aκ (5 μg/mL) to an irrelevant antigen (clone RTK2758, Biolegend) was added to the cultures. CD16 expression on monocytes was then measured.

### Statistical analysis

We used commercial statistical software (JMP 11 system®, SAS Institute Inc., Cary NC, USA). The Wilcoxon rank sum test was used to assess the statistical significance of differences between groups. Correlation between two continuous variables was analyzed using Spearman’s rank correlation coefficient. Dunn’s test was used for multiple comparison procedures. A *p* value <0.05 was considered statistically significant.

## Results

### Clinical characteristics of the patients with RA

Baseline characteristics of the 35 patients are shown in Table 1. A total of 68.6% were RF-positive and 61.8% were ACPA-positive. The mean DAS28-ESR score of the patients was 4.83 ± 0.91. The mean MTX dose at 12 weeks was 10.8 mg/week (8–16). The mean DAS28-ESR decreased from 4.83 at baseline to 3.53 at 12 weeks (*p* < 0.001) (Table 2). Other clinical parameters also significantly decreased, as shown in Table 2.

**Table 1:** Baseline characteristics of patients with rheumatoid arthritis at baseline

	Total (*n* = 35)
Mean age, years	59.80 ± 12.64
Female, *n* (%)	29 (82.9)
Disease duration, months	41.9 ± 76.2
CRP, mg/dL	0.64 ± 0.59
ESR, mm/h	35.7 ± 20.0
DAS28-ESR	4.83 ± 0.91
DAS28-CRP	4.02 ± 0.90
MMP-3 (ng/mL)	100.3 ± 84.8
RF-positive, *n* (%)	24 (68.6)
ACPA-positive, *n* (%)	21 (61.8)
SDAI	18.99 ± 9.22
CDAI	18.35 ± 9.03
HAQ	0.85 ± 0.68

*CRP* C-reactive protein, *ESR* erythrocyte sedimentation rate, *DAS-28* disease activity score in 28-joint count, *MMP-3* matrix metalloproteinase-3, *RF* rheumatoid factor, *ACPA* anti-cyclic citrullinated peptide antibody, *SDAI* simplified disease activity index, *CDAI* clinical disease activity index, *HAQ* health assessment questionnaire

**Table 2:** Changes in disease activity and the proportion of monocyte subsets in patients with rheumatoid arthritis (*n* = 35) at baseline and following 12 weeks of methotrexate treatment

	Baseline	12 weeks	*p* value
Disease activity parameters			
DAS28-ESR	4.83 ± 0.91	3.53 ± 1.25	<0.001
SDAI	18.99 ± 9.87	10.24 ± 7.77	<0.001
CDAI	18.35 ± 9.03	9.84 ± 7.55	<0.001
ESR	35.7 ± 20.0	27.2 ± 23.4	0.0040
CRP	0.64 ± 0.59	0.35 ± 0.59	0.031
Monocyte subsets			
CD14^bright^CD16- monocytes (%)	72.16 ± 9.87	77.57 ± 9.36	0.0042
CD14^bright^CD16+ monocytes (%)	13.96 ± 7.02	10.89 ± 7.25	0.0019
CD14^dim^CD16+ monocytes (%)	8.14 ± 4.84	6.41 ± 4.20	0.0619

Statistical analysis, Wilcoxon rank sum test. *DAS-28* disease activity score in 28-joint count, *SDAI* simplified disease activity index, *CDAI* clinical disease activity index, *ESR* erythrocyte sedimentation rate, *CRP* C-reactive protein.

### Proportions of each monocyte subset

**Fig. 1:**
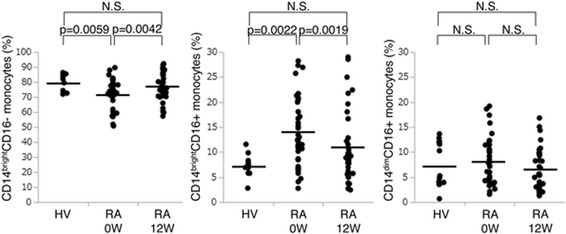
Proportions of CD14^bright^CD16+, CD14^dim^CD16+, and CD14^bright^CD16- monocytes in peripheral blood obtained from patients with rheumatoid arthritis (*RA*) and from healthy volunteers at baseline (*0 W*) and 12 weeks (*12 W*). CD14^bright^CD16-/CD14^bright^CD16+/CD14^dim^CD16+ monocytes were identified by flow cytometry. The proportion of the three subsets of monocytes in patients with RA at baseline and 12 weeks (*n* = 35) was compared with that in healthy volunteers (*HV*) (*n* = 14). Statistical analysis, Wilcoxon rank sum test. Proportions of CD14^bright^CD16- and CD14^bright^CD16+ were significantly different. *N.S.* not significant

Figure 1 shows the three monocyte subsets of peripheral blood cells from patients with RA at baseline and healthy volunteers. The proportion of CD14^bright^CD16+ monocytes in patients with RA was significantly higher than that in healthy volunteers (mean 14.0 ± 7.0% vs. 7.4 ± 2.2%), while that of CD14^dim^CD16+ monocytes did not differ between the two groups (mean 7.3 ± 4.5% vs. 8.1 ± 4.9%). In contrast, the CD14^bright^CD16- population was significantly decreased in patients with RA (mean 72.1 ± 9.9% vs. 79.8 ± 5.7%) compared to healthy volunteers.

After 12 weeks of MTX treatment, the proportion of the CD14^bright^CD16+ population had significantly decreased from 14.0% to 10.9% and that of CD14^bright^CD16- monocytes had significantly increased from 72.2% to 77.6% in the patients with RA, while there was no significant difference in the proportion of CD14^dim^CD16+ monocytes between baseline and 12 weeks (Table 2).

### Association between clinical parameters and monocyte subsets

**Fig. 2:**
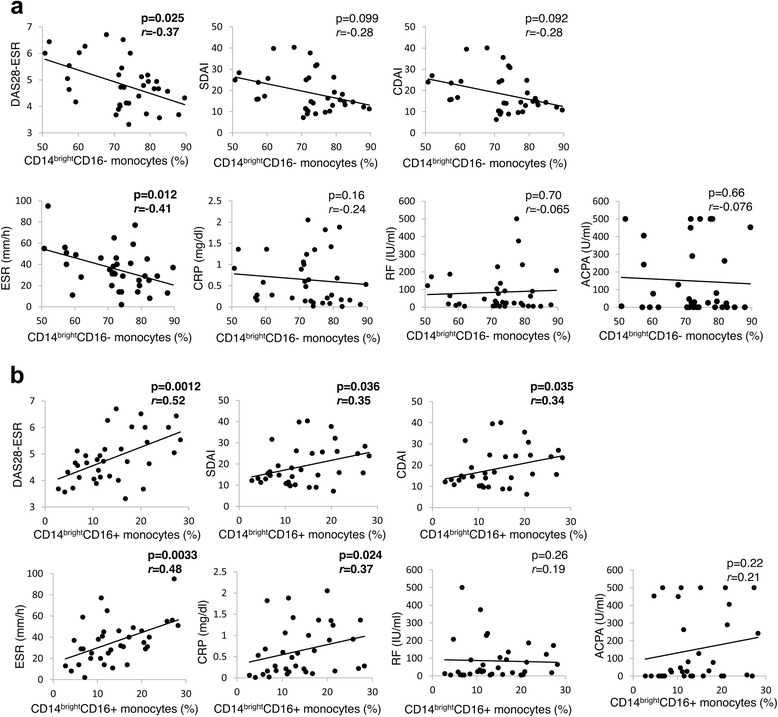
Correlation between the proportion of CD14^bright^CD16-/CD14^bright^CD16+ monocytes and clinical parameters at baseline in patients with rheumatoid arthritis (RA) (*n* = 35). CD14^bright^CD16-/CD14^bright^CD16+ monocytes were identified by flow cytometry. The association between the proportion of CD14^bright^CD16- (a)/ CD14^bright^CD16+ (b) monocytes and clinical parameters is shown. Statistical analysis, Spearman’s rank correlation. r: correlation coefficient. *DAS-28* disease activity score in 28-joint count, *SDAI* simplified disease activity index, *CDAI* clinical disease activity index, *ESR* erythrocyte sedimentation rate, *CRP* C-reactive protein, *RF* rheumatoid factor, *ACPA* anti-cyclic citrullinated peptide antibody

The association between CD14^bright^CD16-/CD14^bright^CD16+ monocytes and clinical parameters is shown in Fig. 2. The proportion of CD14^bright^CD16- monocytes was significantly and negatively correlated (Fig. 2a), while that of CD14^bright^CD16+ monocytes was significantly and positively correlated, with DAS28-ESR at baseline (Fig. 2b). These results indicate that CD14^bright^CD16+ monocytes were positively correlated and CD14^bright^CD16- monocytes were negatively correlated with RA activity. The CD14^bright^CD16+ monocyte subset was also correlated with other parameters such as CDAI, SDAI and CRP. Accordingly, we utilized DAS28-ESR as a representative indicator for the following analysis.

### Association between each monocyte subset and serum cytokine concentration

**Fig. 3:**
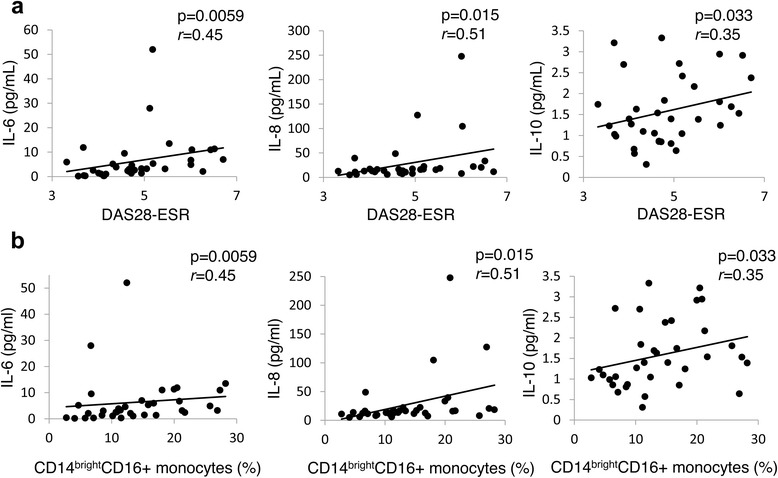
Correlation between serum cytokines (IL-6, IL-8, and IL-10) and the proportion of CD14^bright^CD16+ monocytes at baseline in patients with rheumatoid arthritis (*n* = 35), by 28-joint disease activity score based on erythrocyte sedimentation rate (*DAS28-ESR*). Statistical analysis, Spearman’s rank correlation coefficient. r: correlation coefficient

Among the ten serum inflammatory cytokines, levels of IL-6, IL-8 and IL-10 at baseline were significantly higher in patients with RA than in healthy volunteers (Table 3). These cytokines were positively correlated with DAS28-ESR (Fig. 3a). Moreover, the proportion of CD14^bright^CD16+ monocytes was significantly and positively correlated with serum IL-6, IL-8, and IL-10 (Fig. 3b).

**Table 3:** Serum levels of inflammatory cytokines at baseline in patients with rheumatoid arthritis (RA) and healthy volunteers

	RA (*n* = 35)	HV (*n* = 14)	*p* value
GM-CSF	0.089 (0–0.28)	0 (0–0.45)	0.37
IFN-γ	1.31 (0.87–2.04)	1.17 (0.80–3.19)	0.68
IL-1β	0.027 (0–0.12)	0 (0–0.18)	0.74
IL-10	1.39 (0.98–2.16)	0.95 (0.55–1.29)	0.017
IL-12	0.70 (0.38–1.06)	0.53 (0.21–0.99)	0.21
IL-2	0.19 (0.046–0.30)	0.13 (0–0.22)	0.16
IL-6	3.19 (1.36–6.95)	0.51 (0.35–1.09)	0.0005
IL-8	13.77 (10.81–20.53)	10.90 (5.44–14.21)	0.019
TNF-α	3.75 (2.55–4.61)	3.48 (2.70–4.58)	0.99
M-CSF	164.84 (107.93–266.70)	157.82 (141.32–194.77)	0.82

Data presented as median in pg/mL (interquartile range). *HV* healthy volunteers, *GM-CSF* granulocyte-macrophage colony-stimulating factor, *IFN-γ* interferon-γ, *M-CSF* macrophage colony-stimulating factor. Statistical analysis, Wilcoxon rank sum test

### IL-10 induces surface CD16 expression on monocytes in vitro

**Fig. 4:**
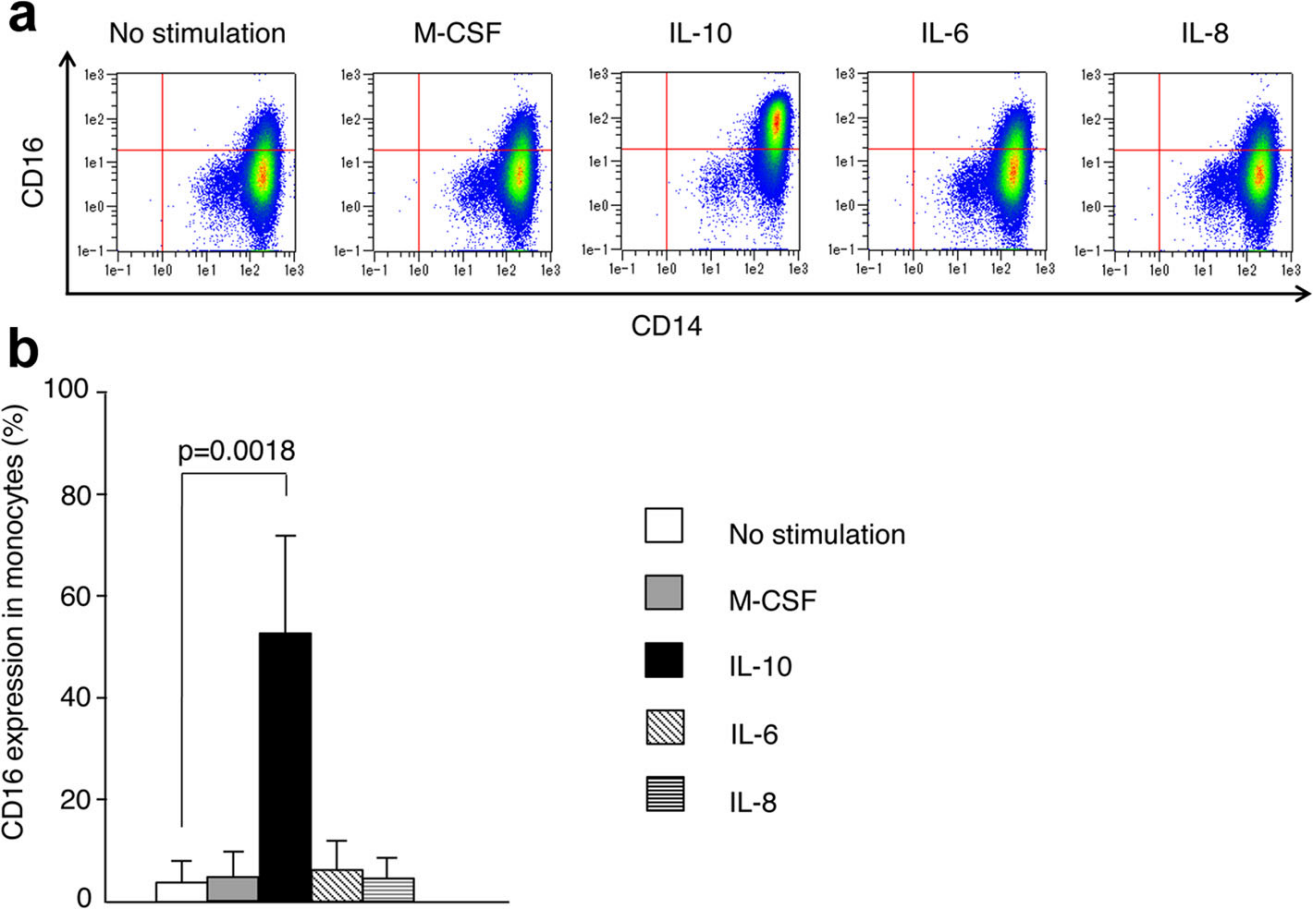
CD16 surface expression by CD14^bright^CD16- monocytes induced by IL-10. CD14^bright^CD16- monocytes from healthy volunteers (*n* = 5) were stimulated with M-CSF (100 ng/mL), IL-10 (25 ng/mL), IL-6 (100 ng/mg), or IL-8 (20 ng/mL) for 14 hours. CD16 expression on CD14^bright^CD16- monocytes was evaluated by flow cytometry. **a** Representative dot plots of CD14 and CD16 expression by monocytes after stimulation with macrophage colony-stimulating factor (*M-CSF*), IL-10, IL-6, or IL-8 for 14 hours. **b** CD16 expression by monocytes in healthy volunteers (*n* = 5). *Bars* show the median ± SD. Statistical analysis, Dunn’s test

To investigate how those cytokines contribute to CD16 expression on monocytes, we isolated CD14^bright^CD16- monocytes from five healthy volunteers and cultured the cells with IL-6, IL-8, IL-10, or M-CSF for 14 hours in vitro. We attempted to stimulate CD16 expression with M-CSF as described in previous reports [12, 17]. Mean purity of the CD14^bright^CD16- subset was 96.8%. Remarkably, the expression of CD16 on the cells was strongly and significantly increased with IL-10 for 14 hours, whereas M-CSF, IL-6, and IL-8 did not induce CD16 expression significantly (Fig. 4a and b). To assess equivalence to CD14^bright^CD16+ cells generated from CD14^bright^CD16- classical monocytes, we analyzed HLA-DR expression on the cultured cells by flow cytometry, because the mean fluorescence intensity (MFI) of CD14^bright^CD16+ monocytes is higher than that of CD14^bright^CD16- monocytes [14, 18]. The MFI of the HLA-DR on CD14^bright^CD16+ cells cultured with IL-10 was higher than that of HLA-DR on CD14^bright^CD16- cells without IL-10 (data not shown), and increased almost as much as that of CD14^bright^CD16+ monocytes in peripheral blood, confirming that ex vivo CD14^bright^CD16+ cells generated from CD14^bright^CD16- monocytes had HLA-DR expression identical to that of native CD14^bright^CD16+ cells.

**Fig. 5:**
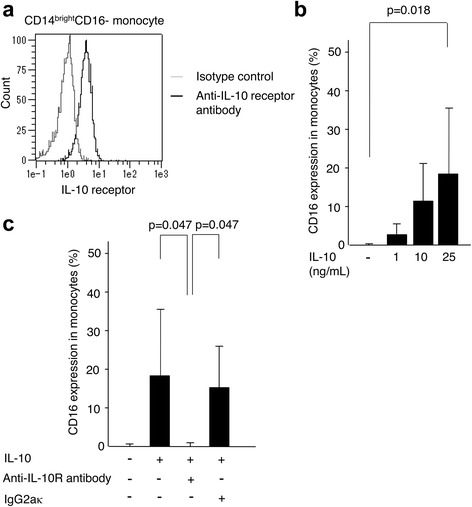
Inhibition of CD16 expression on monocytes by blockade of IL-10 receptors. **a** A representative of mean fluorescence intensity shows surface expression of IL-10 receptors using allophycocyanin (APC)-conjugated anti-IL-10 receptor and rat IgG2aκ antibodies on CD14^bright^CD16- monocytes. **b** CD14^bright^CD16- monocytes from healthy volunteers (*n* = 4), stimulated with various concentrations of IL-10 (0, 1, 10, 25 ng/mL) for 14 hours. **c** CD14^bright^CD16- monocytes from healthy volunteers stimulated with IL-10 (25 ng/mL), anti-IL-10 receptor antibody (5 μg/mL), and/or rat IgG2aκ (5 μg/mL). Effect of IL-10 and anti-IL-10 receptor antibody (5 μg/mL) on CD16 expression of monocytes incubated for 14 hours is shown. The median of CD16 expression in monocytes was 0.06% and 0.13% when incubated with nothing or IL-10 + anti-IL-10 receptor, respectively. *Bars* (**b**, **c**) show the median ± SD. Statistical analysis, Dunn’s test

We then confirmed the surface expression level of IL-10 receptor on CD14^bright^CD16- monocytes. As a result, more than 90% of the cells were IL-10 receptor-positive (Fig. 5a). When CD14^bright^CD16- monocytes were incubated with graded concentrations of IL-10 (1–50 ng/mL), the proportion of CD16 expression on monocytes increased in a dose-dependent manner with 1–25 ng/mL of IL-10 (Fig. 5b). There was almost the same enhanced level of CD16 on the cells when the cells were cultured with 50 ng/mL of IL-10. To confirm the direct effect of IL-10 through the IL-10 receptor, we further conducted a neutralization assay using anti-IL-10 receptor blocking antibody. As a result, the enhanced level of CD16 on the cells (18.3 ± 17.1%, *p* = 0.047) by IL-10 was significantly suppressed by adding anti-IL-10 receptor antibody (0.19 ± 0.19%), whereas isotype control (rat IgG2aκ) did not suppress that enhancement (15.3 ± 10.5%, *p* = 0.047) (Fig. 5c). These results indicate that cell signaling through the IL-10 receptor contributes to regulate CD16 expression on monocytes.

## Discussion

In this study, we demonstrate that circulating CD14^bright^CD16+ monocytes are increased in patients with RA in the active phase and decrease after MTX treatment in a manner that correlates with decreasing disease activity. Moreover, this monocyte subset is associated with expression of inflammatory cytokines in peripheral blood, and the cytokine IL-10, which is increased in patients with RA, induces CD16 expression on monocytes. These results suggest that CD14^bright^CD16+ monocytes play a role in the pathogenesis of RA, and that IL-10 is a key cytokine in the regulation of CD16 expression.

Although an increase in CD14^bright^CD16+ monocytes in patients with RA has been reported [14], the possibility of correlation between CD14^bright^CD16+ monocytes and cytokines in untreated patients with active RA has not yet been investigated. Previous studies have not ruled out any influence of treatment on cytokine and disease activity, because the patients with RA in these studies were not treatment-naïve. Our results support previous observations [14] and show that the proportion of CD14^bright^CD16+ monocytes is increased in treatment-naïve patients with active RA compared to healthy controls, and is positively correlated with disease activity in these patients.

We further showed that the CD14^bright^CD16- population was negatively correlated with RA disease activity. Notably, the proportion of CD14^bright^CD16+ monocytes decreased and that of CD14^bright^CD16- monocytes increased when the patients received MTX treatment.

Though the function of CD14^bright^CD16+ monocytes in RA is not still clarified, this population may be involved in the pathogenesis of RA in accordance with our findings that CD14^bright^CD16+ monocytes decreased after MTX treatment. It has been shown that MTX inhibits inflammatory cytokine production [19] and cell proliferation in vitro, and to induce apoptosis of immune cells [20], but it has not been found to inhibit a specific subset of monocytes. One of the reasons for the decreased proportion of CD14^bright^CD16+ monocytes after MTX treatment was probably secondary to a phenomenon derived from the improvement in RA disease activity.

It is reported that M-CSF plays an important role in the introduction of CD14^bright^CD16+ monocytes. Anti-M-CSF antibody caused a decrease in circulating CD14^bright^CD16+ and CD14^dim^CD16+ monocytes in a clinical trial in two patients with active RA [17]. Moreover, M-CSF and IFN-γ therapy has been found to induce CD16 expression on circulating monocytes in patients with cancer or lymphoma [21]. CD16 expression has been shown to be induced in monocytes with culture of whole PBMCs with M-CSF or IL-10 on CD14^bright^CD16- monocytes in vitro [12]. Although this report may indicate a possible direct role for cytokines, it did not exclude interactions with other cell subsets among the PBMCs. In our study, using highly purified CD14^bright^CD16- monocytes, we clearly showed that IL-10, but not M-CSF, directly induces CD16 expression in CD14^bright^CD16- monocytes. In addition, we proved that the enhancement of CD16 expression on CD14^bright^CD16- monocytes required the interaction with IL-10 and IL-10 receptor by a neutralization assay with anti-IL-10 receptor antibody.

It is well-known that IL-10 plays a crucial role, such as anti-inflammatory and/or pro-inflammatory roles in the pathogenesis of RA. IL-10 has been shown to inhibit production of IL-6, TNF-α, and GM-CSF from immune cells [22], and to enhance B cell differentiation to cells secreting IgG, IgM, and IgA [23, 24], resulting in increased RF and IgG-RF production by B cells in peripheral blood. Moreover, IL-10 is localized to the synovial membrane lining layer, the site of monocyte migration, and inhibits pro-inflammatory cytokines in RA [25]. In this study, we demonstrated that serum IL-10 in patients with RA was significantly elevated compared with healthy volunteers, and was correlated with disease activity.

IL-10 is secreted by many kinds of cells such as T-cells, B-cells, macrophages, dendritic cells, natural killer cells, and monocytes themselves [26–28]. It is reported that CD16 expression on monocytes is maintained by IL-10 production by human naïve CD4+ T cells [29]. The function of CD14^bright^CD16+ monocytes may be regulated by these cells producing IL-10. IL-10 tended to decrease in patients with RA with decreasing CD14^bright^CD16+ monocytes after 12 weeks of treatment. IL-10 may play a role in the induction of CD16 on monocytes in patients with RA.

We note two limitations to our study. First, the number of patients was relatively small, albeit large enough to provide statistically significant data. Second, we did not show that CD14^bright^CD16+ monocytes are directly associated with inflammatory cytokines in RA in vivo. Production of IL-6 and TNF-α in CD14^bright^CD16+ monocytes was not higher than that in CD14^bright^CD16-monocytes (Additional file 1). We thought that CD14^bright^CD16+ monocytes could exert both inflammatory and anti-inflammatory effects, and which effect’s dominance would depend on cells producing IL-10. The functions of these monocytes in RA will need to be clarified in future studies.

## Conclusions

In conclusion, we have shown that CD14^bright^CD16+ monocyte proportions correlate with disease activity in patients with RA, and that CD16^bright^CD14+ monocytes are induced by IL-10 but not by other cytokines upregulated in serum from patients with RA. Our results suggest that CD16^bright^CD14+ monocytes are possibly involved in the pathogenesis of RA and that IL-10 should be a key cytokine that regulates CD16 expression in monocytes.

## Additional file

Additional file 1: Supplemental Method and Figure. (ZIP 314 kb)

## Abbreviations

ACPA: anti-cyclic citrullinated peptide antibody
APC: allophycocyanin
CDAI: clinical disease activity index
CRP: C-reactive protein
DAS-28: disease activity score in 28-joint count
ELISA: enzyme-linked immunosorbent assay
ESR: erythrocyte sedimentation rate
FcγRIII: receptor for immunoglobulin gamma Fc region III
FcγRs: Fcγ receptors
GM-CSF: granulocyte-macrophage colony-stimulating factor
HAQ: health assessment questionnaire
HLA: human leukocyte antigen
IFN: interferon
Ig: immunoglobulin
IL: interleukin
IQR: interquartile range
M-CSF: macrophage colony-stimulating factor
MFI: mean fluorescence intensity
MMP-3: matrix metalloproteinase-3
MTX: methotrexate
PE: phycoerythrin
RA: rheumatoid arthritis
RF: rheumatoid factor
SDAI: simplified disease activity index
TNF: tumor necrosis factor
