# Supplementary figures and images for: CD14brightCD16+ intermediate monocytes are induced by interleukin-10 and positively correlate with disease activity in rheumatoid arthritis
Source: Arthritis Res Ther. 2017 Feb 10;19:28. doi: 10.1186/s13075-016-1216-6 (PMC5301443; doi:10.1186/s13075-016-1216-6)

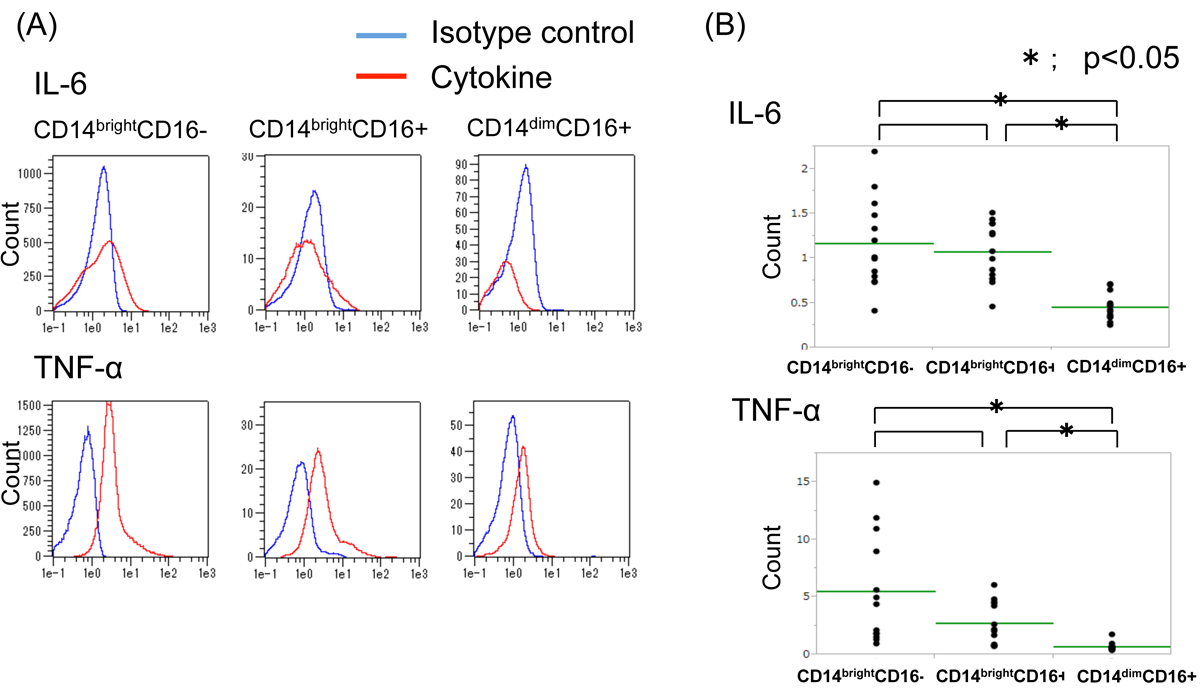

Supplement: Additional file 1: — Supplemental Method and Figure. (ZIP 314 kb) [file 13075_2016_1216_MOESM1_ESM.zip › additional file 1/Supplementary Figure.tif]
