# Supplementary material for: CD14brightCD16+ intermediate monocytes are induced by interleukin-10 and positively correlate with disease activity in rheumatoid arthritis
Source: Arthritis Res Ther. 2017 Feb 10;19:28. doi: 10.1186/s13075-016-1216-6 (PMC5301443; doi:10.1186/s13075-016-1216-6)
Supplement: Additional file 1: — Supplemental Method and Figure. (ZIP 314 kb) [file 13075_2016_1216_MOESM1_ESM.zip › additional file 1/Additional file.doc]

Additional file

**Analysis of intracellular cytokines in monocytes from active RA patients**

Intracellular cytokine staining was performed using the Brefeldin A (GolgiPlug®, BD Biosciences, San Jose CA, USA). Peripheral blood mononuclear cells (PBMCs) were isolated from 11 active RA patients by density gradient centrifugation (Ficoll-Paque®, GE Healthcare, Uppsala, Sweden). PBMCs were cultured at 2 × 10^6^/mL in Roswell Park Memorial Institute medium (RPMI-1640®, ATCC, Manassas, VA, USA) with 10% heat-inactivated fetal bovine serum (MP Biomedicals, Santa Ana CA, USA) for 4 hours at 37°C in a humidified atmosphere containing 5% CO_2_ in the presence of Brefeldin A. Cells were washed twice in MACS buffer (Miltenyi Biotec, Bergisch Gladbach, Germany) and were incubated with Phycoerythrin-Cy7 (PE-Cy7)-conjugated anti-CD14 (clone M5E2, BD Pharmingen, San Diego CA, USA) and V450-conjugated anti-CD16 antibodies (clone 3G8, BD Horizon, San Jose CA, USA) on ice for 15 min. Cells were washed with MACS buffer then fixed by FluoroFix Buffer (Biolegend, San Diego CA, USA). Cells were washed twice in Perm/Wash (Biolegend) solution and were stained with Alexa Fluor 488-conjugated anti-TNF (clone MAb11, Biolegend) and APC-conjugated anti-IL-6 antibodies (clone MQ2-13A5, Biolegend) by incubating for 20 min in 100 μL of Perm/Wash. Stained cells were washed in Perm/Wash solution and resuspended in 250 μL of MACS buffer prior to flow cytometry analysis. The expression levels of cytokines in each monocyte subset were evaluated.

Supplementary Figure legend

**Analysis of intracellular cytokines in monocytes from active RA patients**

(A) Representative image of mean fluorescence intensity shows intracellular TNF-α and IL-6 using Alexa Fluor 488-conjugated anti-TNF-α, Alexa Fluor 488-conjugated mouse IgG1κ, APC-conjugated anti-IL-6, and APC-conjugated rat IgG1κ antibodies in each monocyte subset. (B) Intracellular TNF-α and IL-6 in each monocyte subset from 11 active RA patients. Statistics: Dunn’s test.
